# Outpatient Mental Health Treatment Utilization and Military Career Impact in the United States Marine Corps

**DOI:** 10.3390/ijerph15040828

**Published:** 2018-04-23

**Authors:** Marjan Ghahramanlou-Holloway, Jessica M. LaCroix, Kari Koss, Kanchana U. Perera, Anderson Rowan, Marcus R. VanSickle, Laura A. Novak, Theresa H. Trieu

**Affiliations:** 1 Department of Medical & Clinical Psychology, Suicide Care, Prevention, and Research Initiative, Uniformed Services University of the Health Sciences, Bethesda, MD 20814, USA; jessica.lacroix.ctr@usuhs.edu (J.M.L.); kari.r.koss@gmail.com (K.K.); kanchana.perera.ctr@usuhs.edu (K.U.P.); marcus.r.vansickle.mil@mail.mil (M.R.V.); laura.novak.ctr@usuhs.edu (L.A.N.); theresa.trieu.ctr@usuhs.edu (T.H.T.); 2 School of Psychology and Counseling, Regent University, Virginia Beach, VA 23464, USA; arowan@regent.edu

**Keywords:** mental health treatment, career impact, military, suicide, United States Marines Corps

## Abstract

Service members (SM) are at increased risk of psychiatric conditions, including suicide, yet research indicates SMs believe seeking mental health treatment may negatively impact their military careers, despite a paucity of research examining actual career impacts. This study examined the link between seeking outpatient mental health (MH) treatment and military career impacts within the United States Marine Corps. In Phase 1, a retrospective medical record review of outpatient MH treatment-seeking Marines (*N* = 38) was conducted. In Phase 2, a sample of outpatient MH treatment-seeking Marines (*N* = 40) was matched to a non-treatment-seeking sample of Marines (*N* = 138) to compare career-progression. In Phase 1, there were no significant links between demographic, military, and clinical characteristics and referral source or receipt of career-affecting treatment recommendations. In Phase 2, MH treatment-seeking Marines in outpatient settings were more likely than matched controls to be separated from the military (95.0% versus 63.0%, *p* = 0.002), but no more likely to experience involuntary separation. MH treatment-seeking Marines were more likely to have documented legal action (45.0% versus 23.9%, *p* = 0.008) and had a shorter time of military service following the index MH encounter than matched controls (*p* < 0.001). Clinical, anti-stigma, and suicide prevention policy implications are discussed.

## 1. Introduction

Research has shown that military service members (SM) are at increased risk of psychiatric conditions including depression, post-traumatic stress disorder (PTSD), sleep problems, and suicide [1,2]. Suicide accounted for 28.1% of all deaths that occurred in the United States (U.S.) armed forces in 2013, making it a leading cause of death among military personnel [3]. Rates of suicide among U.S. Marines have been among the highest within the service branches since 2001, increasing to a rate of 21.2 per 100,000 people in 2015 [4,5]. Despite the seriousness of suicide-related thoughts and behaviors, the most recent Department of Defense Suicide Event Report (DoDSER) indicated that only 28% of SMs who had died by suicide and 22% of those who had attempted suicide were documented as having spoken to someone about their potential for self-harm [5]. Disclosure of suicide ideation (SI) or other psychiatric problems is difficult when the consequences, including being forced to undergo a psychological evaluation, are potentially disruptive to the service member’s career [6]. Indeed, SMs were less likely to disclose SI in risk-assessment protocols when informed that their responses could result in referral to a behavioral health provider [7].

Recently, the availability of comprehensive behavioral health care and evidence-based treatments for SMs have increased; however, only 29% of SMs who reported experiencing a mental health problem within the past year also reported seeking mental health services in that same period [1]. Overall, only about 40% of personnel with a perceived need for mental health care actually seek care during their time in service [2,8]. Current military suicide prevention efforts have focused on an “identify and refer” framework where SMs who meet certain warning signs are sent to a mental health provider [9]. Unfortunately, as a side effect of this approach, SMs who disclose suicide-related distress risk a loss of their autonomy (e.g., “suicide watch”), potentially leading to increased stigma and concealment of mental health distress [10]. Pre- and post-deployment health assessments, designed to identify SMs in need of care, can result in referrals to mental health, and SMs who are referred must be assessed before they can deploy or return home [1], potentially hindering the honest self-reporting of psychiatric symptoms. SMs who disclose suicidality or other significant psychiatric problems may also risk notification to their chain of command in an effort to ensure the safety of the member [7]. In a culture that emphasizes resilience and self-reliance, the need to seek help for mental health struggles can be seen as weakness, which may discourage treatment-seeking [2,9]. Indeed, one recent meta-analysis found that the two most commonly endorsed stigma-related barriers to treatment-seeking included “I would be seen as weak” and “My unit leadership might treat me differently” [8]. Additional concerns commonly expressed by SMs regarding the impact of mental health treatment include the belief that unit members might have less confidence in them, it would be embarrassing, and that seeking treatment would harm their careers [8,11].

Although the belief that seeking mental health treatment may negatively impact one’s military career is commonly expressed, the extent to which SMs endorse career-related concerns varies widely, from 12% to 37% in U.S. military samples [1]. In one well-known study, career concerns were endorsed as a barrier to treatment-seeking by 50% of SMs who met criteria for a psychiatric disorder, and by 24% of SMs who did not meet criteria for a psychiatric disorder [11]. Among those who have actually sought behavioral health services, 21% of SMs reported having experienced a negative impact on their careers as a result; this experience was most common among Marines (26%) and Sailors (24%) [12]. In consideration of mental health impact on military service, it is important to note that there are in fact several medical and psychiatric conditions that may contribute to a designation of “not fit” for military service. While each branch of service maintains their own standards of medical fitness, they closely parallel one another and categorize conditions into “fitness” (i.e., ability to do one’s job) and “suitability” (i.e., ability to do one’s job within the military environment). Medical conditions across all domains can impact continued service and lead to separation due to the unique mission of the armed forces. In relation to mental fitness for duty, SMs with conditions such as schizophrenia, bipolar I and II, and recurrent and severe mood disorders that require hospitalization or extended periods of non-deployability are referred to a medical evaluation board for consideration of medical retirement. Conditions such as substance use disorders and severe personality disorders that limit one’s ability to interact effectively within the unit toward mission completion may lead SMs to be considered “not suitable for duty” and they may be referred for an administrative separation from service [13,14]. Further, studies have found that pre-deployment mental health problems and post-deployment conditions such as PTSD may be associated with misconduct that can ultimately lead to punitive discharge [15,16]. Notably, these conditions have relatively low base rates and make up only a small fraction of a percent of those seeking mental health care, suggesting the impact these “known” cases have on stigma is extremely disproportionate.

Empirical data examining the link between mental health treatment-seeking and subsequent military career-related outcomes remains sparse. A retrospective chart review of U.S. Air Force (USAF) personnel indicated that 6% of SMs who sought mental health treatment in 2002 received potentially career-impacting treatment recommendations including duty restrictions (e.g., weapons-bearing status) and retention recommendations (e.g., administrative separation or referral to a medical evaluation board) [17]. Among Army personnel deployed to Afghanistan in 2006 and 2007, 19% of Soldiers who sought mental health treatment received career-impacting recommendations [18], and among USAF SMs seeking treatment in 2010, 29% received career-impacting treatment recommendations [19]. This last study included a prospective, case-controlled comparison of the career trajectories of USAF personnel who did and did not seek mental health treatment. Results indicated that SMs who sought mental health services were more likely to receive a medical board evaluation (16% versus 6%) and were more likely to be discharged from service (24% versus 15%) than matched controls. However, it is important to note that the majority of discharges were honorable; only one instance included a bad conduct discharge. It is also important to note that treatment-seeking SMs were no more likely than controls to experience a change in level of security clearance, consistent with research indicating that less than 0.2% of clearance denials and revocations are due to mental health issues [20]. In all three studies mentioned above, SMs who self-referred to treatment were less likely than SMs who were encouraged by their superiors or command-directed to receive career-impacting recommendations [17,18,19]. While these studies suggest that commanders are appropriately using command-directed evaluations to identify SMs who have identifiable duty limitations, they also suggest that early and voluntary care is critical for suicide prevention and improved mental health, as well as operational readiness and lessened career impact for SMs.

### Aims and Hypotheses

This study aimed to replicate and expand upon previous studies that had examined mental health treatment-seeking patterns and military career-related impact among USAF and Army SMs [17,18,19] by investigating these variables among United States Marine Corps (USMC) personnel. This study was conducted in two phases: Phase 1—retrospective chart review, and Phase 2—prospective analysis. The specific aims in Phase 1 were: (1) to describe demographic, military, and clinical characteristics of a sample of Marine outpatients who had received mental health services at military treatment facilities; (2) to compare Marine outpatients (seeking outpatient mental health services) who had self-referred, who were referred by superiors, and who were command-directed to receive a psychological evaluation on demographic, military, and clinical characteristics; (3) to compare Marine outpatients who had received potentially career-impacting provider recommendations versus those who had received non-career-impacting provider recommendations on demographic, military, and clinical characteristics; and (4) to compare Marine outpatients with a documented history of suicide ideation (SI) and/or suicide attempt (SA) versus Marine outpatients without suicide-related documentation on demographic, military, and clinical characteristics. The specific aim in Phase 2 was to compare the career trajectories of Marine outpatients who had received care at military mental health facilities to those of a matched sample of personnel who had not received such care. Consistent with prior research [17,18,19], we expected that Marine outpatients who self-referred would be less likely to receive career-impacting provider recommendations. We also expected that compared with matched controls, Marines who received care at military treatment facilities would be more likely to experience disruptions to their military careers [19].

## 2. Materials and Methods

### 2.1. Phase 1: Retrospective Chart Review

#### 2.1.1. Participants and Procedures

A random sample of 44 electronic medical records (EMRs) were coded for inclusion in the current study. Originally, 80 EMRs were selected from all Marines who sought outpatient mental health services (i.e., initiated outpatient behavioral health services) from any Navy treatment facility (e.g., naval hospitals, naval branch health clinics) between 1 January 2009 and 31 December 2010. The larger random sample was first identified by the EpiData Center (EDC) at the Navy and Marine Corps Public Health Center; however, due to funding limitations, a smaller random sample of 44 were identified for study inclusion. After obtaining written approval from the local Institutional Review Board (IRB), the Human Research Protection Office (HRPO), and Tricare Management Activity (TMA), the 44 EMRs were reviewed via the Armed Forces Health Longitudinal Technology Application (AHLTA), the electronic documentation system for the Department of Defense (DoD) medical providers. Four of the 44 records were not coded due to a lack of intake information, and two of the medical records were coded, but excluded from Phase 1 due to having an unknown referral source. Therefore, the final sample used for the Phase 1 analyses consisted of 38 medical records of U.S. Marine Corps personnel. Coding was conducted by trained bachelor’s and master’s level research staff members, under the supervision of a doctoral level psychologist utilizing a coding manual developed for this project. Training consisted of reviewing 3 EMRs and conducting independent coding. Variables of interest included demographic (e.g., age, gender), military (e.g., rank), and clinical (e.g., psychiatric disorders, history of suicidality) characteristics, referral source, and documented provider recommendations.

#### 2.1.2. Measures

Psychiatric disorders: Psychiatric disorders were identified by recording the primary ICD-9 diagnostic code documented by the mental health provider and categorizing using *The Diagnostic and Statistical Manual of Mental Disorders—Fourth Edition (Text Revised)* (DSM-IV-TR) [21]. Psychiatric disorders were coded as (1) no diagnosis, (2) V-code, (3) adjustment disorder, and (4) other Axis I diagnoses. The presence of multiple Axis I diagnoses resulted in categorization being made based on the one that was the primary focus of treatment.

Outpatient history: Outpatient history included any documented history of outpatient mental health care in the military received between entry into service and reviewed at intake.

History of suicidal ideation (SI) and/or suicide attempt (SA): If a Marine’s EMR contained any documented history of SI and/or SA, including those that occurred prior to mental health intake, they were coded as having a history of SI/SA. SI and SA were collapsed into one category due to the small number of records reviewed and the small number of Marines with documented history of SI and/or SA.

Treatment status: Treatment status was coded by tracking the mental health visits related to the identified course of treatment. Termination summaries were often used in order to determine whether the patient completed the course of treatment or dropped out before completion. Additionally, records were reviewed for details about the follow-up treatment plan to determine whether or not treatment was completed or if further follow-up was required. Treatment status was coded as completed, incomplete due to mission-related reasons (e.g., being reassigned to another duty station, being deployed), incomplete due to retirement or separation, or incomplete due to unknown reasons (e.g., SM did not follow-up).

Referral source: Referral status was determined based on intake paperwork or intake clinical notes, as documented in the EMR. Consistent with previous research [17,18,19], referral source was coded as self-referred, superior-encouraged, or command-directed. Self-referral was coded when a Marine sought mental health services on his or her own. Superior-encouraged referral refers to SMs whose superiors (e.g., first line supervisors) encouraged them to seek help from mental health, without formally directing them to receive an evaluation. Command-directed referrals were coded for Marines when they were mandated by leadership with command authority to be evaluated for concerns related to safety, fitness or suitability for duty, or judgment, reliability, or stability concerns. An important distinction should be made between “superior-encouraged” and “command-directed”. Specifically, “superior-encouraged” appointments are still voluntary and presented as such throughout the informed consent process, ensuring Marines know they are not required to attend, whereas a command-directed evaluation is mandatory for the member.

Provider recommendations: All patient records were reviewed to determine whether any contact was made with the commander, and what type of recommendation was documented in the patients’ records. For ease of analysis and interpretation, recommendations were classified as “career-affecting” or “non-career-affecting”. In line with Rowan and Campise [17], career-affecting recommendations were considered to be those that resulted in duty restrictions or limitations (e.g., removal of weapons bearing status, duties not to include flying (DNIF), deemed not deployable, etc.), or a recommendation of medical or administrative discharge from the service. Non-career-affecting recommendations were considered to be those that included a return to duty recommendation, supportive recommendations including modification of hours or assistance meeting personal needs, and treatment recommendations. Even though supportive recommendations occasionally involved contact with command, they were coded as non-career impacting because contact was unlikely to negatively affect the SM’s desired duty status.

#### 2.1.3. Statistical Analysis

In Phase 1, bivariate analyses using analysis of variance (ANOVA), and Pearson’s chi-square (χ^2^) tests were conducted to evaluate differences in demographic, military, and clinical variables between referral sources (self-referred, superiors-encouraged, command-directed) and recommendation type (career-related, non-career-related). All statistical analyses were made using the Statistical Analysis System, SAS, version 9.4 (SAS Institute, Inc., Cary, NC, USA) [22] where a *p* < 0.05 (two-tailed) was considered statistically significant.

### 2.2. Phase 2: Prospective Case-Control Comparison

#### 2.2.1. Participants and Procedures

Phase 2 of the study involved pairing each of the coded EMR cases (*N* = 40) with 4 controls cases matched on rank, time in grade, and military occupational specialty (MOS), resulting in a total of 160 controls. Matching was completed by the Armed Forces Health Surveillance Center (AFHSC).

A total of 200 SSNs (40 cases and 160 controls) were provided by the AFHSC directly to the Headquarters Marine Corps Manpower Information Systems Division (MI), to obtain career-related variables for the 5.5-year timeframe from 1 January 2009 to 31 August 2014. Data from all three sources (i.e., EMRs, AFHSC, MI) were subsequently merged by the AFHSC, and returned to our research team in de-identified format. Matched control Marines who had any documented contact with inpatient and/or outpatient mental health services were excluded from analyses (*n* = 22). Thus, between-group comparisons are representative of 40 treatment-seeking cases and 138 non-treatment-seeking controls.

Military career-related variables provided by MI included record status, separation code, security classification, and the endorsement of any legal action. Record status is a system-generated code to indicate the duty status of the Marine’s record (e.g., active, separated, awaiting join, on orders, transferring). Separation code provides information on the reason for the Marine’s separation from active duty. Codes were dichotomized into voluntary separation (e.g., interdepartmental transfer, disability with severance pay, in lieu of trial by court martial) and involuntary (e.g., misconduct, personality disorder, court martial, alcohol rehabilitation failure, reduction in force). Number of years between the identified behavioral health encounter and date of separation was also measured. Security classification refers to security-clearance status including secret/top secret/sensitive compartmented information (SCI), denied clearance, no status, or revoked/loss of jurisdiction. Legal action refers to any history of documented military legal charges (e.g., non-judicial punishment (NJP), summary courts martial (SCM), special courts martial (SPCM), general courts martial (GCM)).

#### 2.2.2. Statistical Analysis

In Phase 2, ANOVA, and Pearson’s chi-square (χ^2^) tests were conducted to evaluate group differences in career-related variables among the treatment-seeking cases, and matched non-treatment-seeking controls. We conducted conditional logistic regression using PROC LOGISTIC with the STRATA command embedded into the coding schema. This method of analysis was chosen to take into account the matched case to control study design, where the observations in the control group are no longer independent, and hence the need for stratification; odds ratios (ORs) were used to report these comparisons.

## 3. Results

### 3.1. Phase 1: Retrospective Chart Review

Demographic, military, and clinical characteristics for the treatment-seeking Marines (*N* = 38) are presented in Table 1. Overall, participants were on average 23.3 years old (standard deviation (*SD*) = 4.2 years), predominantly male (89.5%), Caucasian (68.6%), and single (62.2%). For most (91.7%), the highest level of education was a high school diploma or equivalent. The majority of Marines were junior enlisted (66.7%).

Clinical characteristics indicate that most Marines were diagnosed with other Axis-I disorders (71.1%), which included substance abuse (40.7%), mood (33.3%), anxiety (18.5%), and other (7.4%) disorders (e.g., pain, sleep). Almost two-thirds of the sample (63.2%) did not complete treatment (47.4% due to mission-related reasons, 10.5% for unknown reasons, and 5.3% retired or separated). Of the sample of 38 Marine outpatients, 12 (31.6%) had a documented history of SI/SA (26.3% SI, 5.3% SA), and 26 (68.4%) had no documented history. No significant differences between Marines with or without a history of SI/SA were found on any demographic, military, or clinical variables.

#### 3.1.1. Referral Source

Analyses based on referral source are also presented in Table 1. Marines were more likely to be self-referred (50.0%) than superior-encouraged (36.8%), or command-directed (13.2%). Participants who endorsed a history of seeking outpatient treatment were significantly more likely to be self-referred (47.1%) or command-directed (80.0%) in comparison to those who were superior-encouraged (*p* = 0.033). However, this difference becomes non-significant after accounting for Type I error using the Bonferroni correction. Referral source did not significantly differ between Marines with or without a history of suicide ideation/attempt. Due to the small number of Marines who were command-directed, analyses were rerun comparing those who self-referred to those who were superior-encouraged or command-directed, in order to determine if there were any differences between individuals with or without command/supervisor involvement. Results indicate no significant differences between referral source and demographic or clinical characteristics.

#### 3.1.2. Provider Recommendations

Nearly half (44.7%) of Marines received a career-affecting recommendation from the behavioral health provider (Table 2). No demographic, military, or clinical variable was significantly associated with receipt of career-impacting treatment recommendations. However, self-referral was higher (61.9%) among Marines who did not receive career-affecting treatment recommendations compared to Marines who did receive career-affecting treatment recommendations (35.3%). Nonetheless, this difference was not statistically significant.

### 3.2. Phase 2: Prospective Case-Control Comparison

Prospective data through August 2014 were obtained for both mental health treatment-seeking Marines and matched controls in order to determine whether career-related differences were significant between the groups. The data obtained from Phases 1 and 2 of this study could not be compared on demographic, military, or clinical characteristics due to the de-identified nature of the data provided by the AFHSC.

Table 3 presents the variables related to separation from service provided by Headquarters Marine Corps MI, as it is stored in their personnel database. According to the record status code as of August 2014, 5.0% of treatment-seeking Marines and 32.6% of matched controls remained on active duty. The majority of the treatment-seeking group (95.0%) had separated from the Marine Corps, compared to 63.0% of matched controls. This difference was statistically significant, OR = 10.16, *p* = 0.002. Treatment-seeking Marines were more likely than control Marines to separate voluntarily (34.2% versus 14.9%), and were less likely to separate due to completion of required active service/sufficient service for retirement (26.3% versus 51.7%), OR = 0.19, *p* = 0.010. Treatment-seekers and controls were similarly likely to experience involuntary separation. Treatment-seeking Marines were also significantly more likely than non-treatment seeking Marines to experience legal action (45.0% versus 23.9%), OR = 2.84, *p* = 0.008, and had a shorter average time of military service following the initial mental health encounter (1.5 years versus 2.1 years), *t*(175) = 6.322, *p* < 0.001.

Exploratory analyses indicated that not only was treatment seeking associated with legal action, legal action was also associated with separation, OR = 3.95, *p* = 0.011. Overall, among Marines experiencing legal action (*n* = 127), 84.3% were ultimately separated from service. Among Marines who did not experience legal action (*n* = 51), only 64.6% were ultimately separated from service. In multiple logistic regression analyses predicting separation from service, seeking mental health care remained a significant predictor of separation after controlling for legal action, OR = 9.82, *p* = 0.002. Legal action also remained a significant predictor of separation after controlling for treatment-seeking status, OR = 2.42, *p* = 0.047, indicating that the link between treatment seeking and subsequent separation from service is not completely explained by legal action.

## 4. Discussion

This study examined the relationship between outpatient mental health treatment and career progression within the U.S. Marine Corps (USMC). In Phase 1, retrospective medical record review was conducted for a sample of Marines who sought mental health treatment from 2009 through 2010 to identify demographic, military, clinical and referral characteristics associated with receipt of career-affecting treatment recommendations. Phase 1 of this study was a first effort towards replicating findings reported in samples of USAF and Army personnel [17,18,19] within a USMC sample, and towards understanding the potential association between referral source and receipt of career-affecting provider recommendations. In an effort to expand upon the Phase 1 methodology and evaluate the possible long-term impact of receipt of mental health care on one’s military career, Phase 2 of the current study collected prospective data through August 2014 for Marines seeking outpatient mental health services and a matched sample of non-treatment-seeking controls.

### 4.1. Phase 1: Retrospective Chart Review

In Phase 1, approximately half of treatment-seeking Marines received potentially career-affecting treatment recommendations. There were no significant links between demographic, military, and clinical characteristics, including history of suicidality, and referral source or receipt of career-affecting treatment recommendations. Referral source was not significantly associated with receipt of career-affecting treatment recommendations in the current study; however, it is important to note that the majority of Marines—nearly two-thirds—who did not receive career-affecting treatment recommendations self-referred to treatment. In contrast, among Marines who did receive career-affecting recommendations, only one-third self-referred to treatment. It is also important to note that provider recommendations include short-term impacts that may not have long-term career impacts (e.g., temporary removal of weapons bearing status or security clearance). This study used a conservative approach, such that duty restrictions or change in duty status may be perceived by the SM as truly career impacting at that time, yet we may not be able to determine the ultimate impact of these recommendations on an SM’s overall career based on this study design. Many of the current findings differ from previous studies in other branches of military service. For example, Rowan and Campise’s [17] retrospective review of USAF personnel seeking behavioral health treatment found that Airmen who self-referred for mental health care versus those who were command-directed had less significant documented Axis I psychiatric diagnoses, were less likely to exceed the limits of confidentiality, and were less likely to experience career-impacting recommendations. In a retrospective review of Army personnel seeking mental health treatment while deployed, investigators found that self-referred SMs were less likely to receive career-affecting recommendations or have contact made with their command [18]. Similarly, Ghahramanlou-Holloway and colleagues [19] found that after controlling for demographics and clinical characteristics, self-referred USAF personnel were less likely to experience career-affecting recommendations compared to superior-encouraged personnel. In addition to being underpowered, a number of factors, including the fact that our study was conducted within the USMC as opposed to the USAF or Army, may explain the inability to replicate prior results. Thus, it could be the case that the three populations of military personnel are inherently different, that the documentation practices of providers are different, and/or that the true “career impact” of seeking mental health care is different between the three branches of military service. Further, it is possible that these findings may be an anomaly due to small sample size, and merit replication in a larger sample.

This study uniquely examined the role of documented suicidality in receipt of potential career-related recommendations. Of additional interest was whether the history of suicide ideation and/or attempt differed based on demographic, military, clinical, or referral characteristics. Results indicated that a history of suicidality was not associated with receipt of career-affecting treatment recommendations. The small number of Marines with a history of SI/SA—as well as our small overall sample—limited our ability to make meaningful statistical comparisons regarding other variables.

### 4.2. Phase 2: Prospective Case-Control Comparison

Phase 2 of the study was designed to capture potential long-term outcomes on career-related variables, including military separation and military legal charges. Results from Phase 2 indicate that Marines who sought mental health treatment were more likely to be separated from the service, and at an earlier date compared to control Marines. Marines who sought mental health treatment were also more likely to experience legal charges than control Marines. However, treatment-seeking Marines were equally likely as control Marines to be involuntarily separated. Treatment-seeking Marines were in fact more likely than control Marines to be voluntarily separated, and less likely than control Marines to be separated due to completion of required active service/sufficient service for retirement. These findings are not necessarily surprising in light of other reports regarding attrition rates in the military. While causality cannot be inferred, mental health problems are a documented risk factor for leaving military service. For example, in the aforementioned prospective analysis of USAF SMs, individuals who sought mental health treatment were three times more likely to receive a medical board evaluation and two times more likely to be discharged from the USAF than non-treatment-seeking controls [19]. Prior research conducted within the Australian Navy also indicates that mental health problems are linked with separation—the majority of individuals with a psychiatric diagnosis left military service within the first year of symptom onset [23]. Similarly, a review of U.S. Army and Marine Corps post-deployment health assessment data indicated that, within a year after deployment, individuals who met criteria for a psychiatric disorder were significantly more likely than individuals who did not meet criteria to separate from military service for any reason [24]. More recently, a study examining rates of early separation among deployed Army, Navy, and Marine Corps SMs found that rates of early separation for individuals treated for mental disorders were twice as high as those treated for non-combat physical injuries, and three to five times higher than a random sample [16]. Although separation includes voluntary as well as involuntary reasons, the current study findings replicate these earlier studies that have identified earlier career separations for those with mental health concerns. Indeed, individuals with mental health problems may be more likely to seek mental health treatment and may also be more likely to be voluntarily or medically separated from service. These findings should not be interpreted such that the receipt of mental health treatment, in isolation, results in earlier separation from service, especially involuntary separation.

Career-related impacts of mental disorders may be accounted for by a higher rate of voluntary separation, greater disease chronicity and severity, and/or by a variety of behavioral problems including misconduct, legal problems, unauthorized absences, and alcohol/drug-related problems. Hoge and colleagues [25] found that 17% of all Soldiers hospitalized for mental disorders were involuntarily separated due to misconduct and other legal problems, accounting for one quarter of all separations among these Soldiers. Within our USMC sample, treatment-seeking Marines were more likely than non-treatment-seeking Marines to experience legal action, but the link between treatment-seeking and separation remained significant even after adjusting for experiences of legal action. This suggests that experiencing legal actions alone, presumably for misconduct, cannot account for the rates of military separation observed among Marines with mental health care contact. Rather, nearly two-thirds of treatment-seeking Marines who separated from service after visiting mental health separated voluntarily or after completion of required service.

### 4.3. Limitations

This study has several limitations. First, the sample for this study were exclusively USMC personnel; therefore, results may not generalize to other branches of service. Second, given the relatively small sample size in Phase 1 of this study, findings and comparisons to previous reports must be interpreted with much caution. Due to time and funding limitations, only 40 cases were coded; thus, the analyses were underpowered to detect a true effect. Third, Phase 1 of this study was retrospective in nature. Charts reviewed for this project were gathered from a centralized EMR, which required sifting through numerous seemingly unrelated medical visits in order to code for treatment and subsequent recommendations related to mental health care. In some instances, seemingly unrelated medical visits could have contributed to separation. Additionally, psychiatric diagnoses could have occurred at intake or during the course of treatment, depending on available documentation within the EMR. This has relevance for diagnostic accuracy, as it can take multiple sessions to determine the appropriate psychiatric diagnoses. Finally, documentation practices between military treatment facilities and clinical providers varied and, in some cases, did not provide information related to the variables of interest for this project. For example, termination summaries were used to determine whether or not treatment was completed; however, treatment terminations may not always be documented as a separate summary in the EMR, and may instead be outlined in combination of the subjective and plan sections of a note.

Despite these limitations, to our knowledge, this is the first study that has empirically examined factors associated with receipt of career-affecting mental health care treatment recommendations and compared career trajectories of treatment-seeking and non-treatment-seeking Marines. Research findings, to date, have shown that 37.7% of Marine personnel hold a belief that seeking mental health treatment would damage a person’s military career, and that 26.2% of Marines who had sought treatment in the past year reported it having a negative effect on his/her career [12]. Regardless of whether or not a true effect exists, Marines *believe* that a true effect exists—providing a significant barrier to care. A recent RAND Corporation study [26] identified 209 policies within the DoD that potentially influence stigma, demonstrating the importance of studies such as this to empirically answer questions about career impacts and provide data to inform SMs and policy makers of the potential effects of seeking mental health care in the military. Of additional relevance to mental health and military service, the DoD is considering a new policy requiring medical or administrative separation for members who are non-deployable for a period greater than 12 months [27].

### 4.4. Implications

Several clinical and policy implications within the USMC and Department of Defense (DoD) can be gleaned from this study. Findings from this and other studies suggest that concerns about potential military career impacts associated with seeking mental health care cannot be dismissed or easily answered. The mission of the DoD necessarily places medical restrictions on the members within it; however, this process is complicated and may not be easily understood by service members, contributing to confusion and potential stigma. SMs may require assistance to understand available treatment options and make informed treatment decisions while considering the scope of and exceptions to confidentiality associated with different treatment options. Anti-stigma campaigns within the DoD should provide high-quality training on the potential benefits of treatment and limits to confidentiality to frontline supervisors, chaplains, and mental health providers.

In line with clinical recommendations from the Air Force Guide for Suicide Risk Assessment, Management, and Treatment [28], findings from this study within a Marine sample suggest that mental health providers serving the USMC community should clearly inform patients, as early in the therapeutic relationship as possible, about the degree to which disclosure to command may be necessary or indicated. Indeed, it is ethically required to discuss limits of confidentiality at the earliest possible time while establishing a therapeutic relationship. In cases where imminent risk has been determined (e.g., serious risk of self-harm, harm to others), mental health providers should do as much as possible to ensure the safety of the service member and appropriately document the rationale for steps taken, including procedures for informing command or others as needed. Confidential and non-clinical services provided by the DoD (e.g., military chaplain services, Military OneSource) provide SMs with additional options for seeking help, and may be particularly useful for non-medical counseling. Indeed, previous research has indicated that SMs who have sought mental health treatment on their own have lower odds of receiving career-affecting recommendations, potentially because they sought care at an early stage before negative career impacts were observed [17,18,19,29]. Although limited by the small sample size and lack of statistical power, results of the current study suggest that Marines who self-refer to mental health treatment, presumably before problems escalate, may also be less likely to receive potentially career-impacting treatment recommendations. Having said that, it is necessary for the DoD to provide military chaplains and non-medical counselors with training to address stigma and potential career impacts associated with seeking mental health care to help ensure SMs receive a higher level of care when indicated. Policy makers can use data from this and similar studies to potentially encourage early help seeking, which may be more beneficial and result in less significant career impacts as opposed to avoiding help-seeking.

Marines who experience legal charges during their service potentially represent a segment of the population in strong need of psychological care, particularly given that legal trouble is an identified suicide risk factor [30]. These individuals are likely to experience a number of cumulative stressors, yet may hesitate to seek behavioral health services, especially due to fears of exacerbating their pre-existing or concurrent occupational functioning problems. Marine Corps policies that address perceived stigma within this subgroup may be helpful to consider, and additional research examining the associations between legal actions, mental health treatment, mental health outcomes, and military separation may provide useful information to inform such policies.

Marine leadership has maintained a continued and sustained effort in examining and addressing the problem of mental health stigma. Increased communication about confidentiality and receipt of mental health services may help to aid this effort. Specifically, in voluntary appointments, the provider starts from a position of “no contact” with the member’s command. Barring the presence of imminent risk to self or others, significant fitness or suitability for duty concerns, or potential for severe detriment to mission, the provider is not required to contact command as limits of confidentiality have not been reached. In cases of command-directed evaluations, providers start from a position of contact. In these cases, commanders are required to demonstrate that a significant concern exists for the member’s judgment, reliability, stability, safety, suitability, or fitness for duty. Members are briefed on the DoD “no stigma” policy by their commanders prior to an appointment being established and they are briefed on the limits of confidentiality to these appointments by their provider. Providers performing command-directed evaluations are expected to answer the commander’s concerns using the minimal amount of information necessary [31,32]. As such, a self-referral to mental health treatment provides the greatest level of confidentiality for the member and allows for treatment prior to the worsening of symptoms that likely contribute to reduced suitability or fitness for duty, legal concerns, and command involvement. There are in fact many conditions, related to medical and behavioral health, that are not suitable for military service. Individuals with these conditions typically experience hardship in adapting and limit the level of functioning of their units. In these cases, separation from service is in the best interests of both the member and the service. Therefore, understanding the unique circumstances of each service member, validating his or her concerns about military career outcomes, and providing education about different available options appear to be the best courses of action for maximizing favorable outcomes.

### 4.5. Recommendations for Future Research

Future research should consider many of the limitations of this study. Given the small sample size for the retrospective phase of this study, future research should employ a larger sample size. The strongest recommendation for future research to advance USMC suicide prevention and anti-stigma programmatic efforts is to utilize a mixed methods approach consisting of survey methodology, focus groups, and confidential interviews in order to best understand the concerns associated with seeking mental health care from a military treatment facility. It is important that research within the Marine Corps focuses on personnel from different military occupational specialties within air, ground, and logistics, such that the unique concerns of each subgroup are best captured. In addition, future research should measure and compare help-seeking attitudes between officers, non-commissioned officers (NCOs), and junior enlisted Marines. It may be that frontline supervisors are well-trained to refer distressed Marines for help, but they themselves may not hold positive attitudes about seeking mental health care when in need. Therefore, it is possible that junior Marines are less likely to seek mental health care if they do not have solid role models who demonstrate the strategies needed to take care of one’s psychological health. Having individuals within positions of leadership openly acknowledge and discuss their own journeys to psychological fitness can be invaluable for a young Marine.

## 5. Conclusions

This study provides initial empirical evidence of the association between seeking outpatient mental health care within the military system and potential military career impacts within the Marine Corps. Findings suggest that roughly half of treatment-seeking Marines receive potentially career-affecting treatment recommendations, and treatment-seeking Marines are more likely than non-treatment seeking Marines to separate from military service. Although treatment-seeking Marines were more likely to experience legal action, legal action alone did not explain the greater rate of military separation. Findings suggest that concerns about career impact and limits to confidentiality should be discussed and taken into consideration when recommending treatment options to Marines, and that certain subgroups of Marines may experience more significant career-related impact when seeking mental health care, possibly related to issues precipitating the seeking of care. Early mental health treatment may be especially important to address issues before they have the potential to negatively affect one’s military career. Future research is needed to better inform DoD and USMC psychological fitness, anti-stigma, and suicide prevention initiatives in order to provide optimal mental health treatment and support to Marines while ensuring the strength and readiness of the force.

## Figures and Tables

**Table 1:** Phase 1: Demographic and clinical characteristics of United States Marine Corps mental health treatment-seekers (*N* = 38) *.

Demographic Characteristics ^1^	Total	Referral Source			*p* ^2^
		Self	Superior-Encouraged	Command-Directed	
	(*N* = 38)	(*n* = 19)	(*n* = 14)	(*n* = 5)	
Age, mean (standard deviation (SD)), years	23.3 (4.2)	24.6 (5.2)	22.1 (2.7)	21.4 (1.7)	0.140
Gender					
Female	4 (10.5)	2 (10.5)	1 (7.1)	1 (20.0)	0.605
Male	34 (89.5)	17 (89.5)	13 (92.9)	4 (80.0)	
Race					
White/Caucasian	24 (68.6)	11 (61.1)	10 (83.3)	3 (60.0)	0.467
All others	11 (31.4)	7 (38.9)	2 (16.7)	2 (40.0)	
Marital status					
Single	23 (62.2)	9 (50.0)	10 (71.4)	4 (80.0)	0.327
Married	14 (37.8)	9 (50.0)	4 (28.6)	1 (20.0)	
Education					
4-year degree	2 (8.3)	1 (8.3)	1 (12.5)	0	1.000
HS diploma or equivalent	22 (91.7)	11 (91.7)	7 (87.5)	4 (100.0)	
Military rank					
Junior enlisted (E1-E3)	22 (66.7)	9 (60.0)	9 (69.2)	4 (80.0)	0.712
NCO (E4-E5)	11 (33.3)	6 (40.0)	4 (30.8)	1 (20.0)	
Clinical characteristics ^1^					
Inpatient history					
No	32 (91.4)	14 (82.4)	13 (100.0)	5 (100.0)	0.358
Yes	3 (8.6)	3 (17.7)	0	0	
Outpatient history					
No	21 (60.0)	9 (52.9)	11 (84.6)	1 (20.0)	0.033
Yes	14 (40.0)	8 (47.1)	2 (15.4)	4 (80.0)	
History of SI/SA					
No	26 (68.4)	13 (68.4)	9 (64.3)	4 (80.0)	1.000
Yes	12 (31.6)	6 (31.6)	5 (35.7)	1 (20.0)	
Diagnostic category					
No diagnosis	2 (5.3)	2 (5.3)	0	0	0.322
V-code	2 (5.3)	0	2 (14.3)	0	
Adjustment disorder	7 (18.4)	3 (15.8)	4 (28.6)	0	
Other axis-I disorder	27 (71.1)	14 (73.7)	8 (57.1)	5 (100.0)	
Treatment status ^3^					
Completed treatment	14 (36.8)	8 (42.1)	4 (28.6)	2 (40.0)	0.811
Incomplete	24 (63.2)	11 (57.9)	10 (71.4)	3 (60.0)	

* Data presented as No. (%), except as noted. ^1^ Due to unreported data, some variables will not have the full sample of 38 participants. ^2^ Obtained using analysis of variance (ANOVA) for continuous variables or χ2 for discrete variables (Fisher’s exact test was used for cell counts < 5). ^3^ Incomplete treatment status reasons include: “mission-related” (*n* = 18: 9 self-referred, 7 superiors-encouraged, 2 command-directed), “reason unknown” (*n* = 4: 1 self-referred, 2 superiors-encouraged, 1 command-directed), and “retired/separated” (*n* = 2: 1 self-referred, 1 superiors-encouraged). Due to the small cell counts, data were dichotomized into “completed” or “incomplete”. Note. NCO = non-commissioned officer; SI = suicide ideation; SA = suicide attempt.

**Table 2:** Phase 1: Results of univariate logistic regression predicting career-affecting recommendation following outpatient behavioral health treatment (*N* = 38) *.

Demographic Characteristics ^1^	Total	Recommendation Type		OR (95% CI)	*p*
		Non-Career Affecting	Career Affecting		
	(*N* = 38)	(*n* = 21)	(*n* = 17)		
Age, mean (SD), years	23.3 (4.2)	23.4 (4.5)	23.1 (4.0)	0.98 (0.84–1.15)	0.820
Gender					
Female	4 (10.5)	3 (14.3)	1 (5.9)	1.00	
Male	34 (89.5)	18 (85.7)	16 (94.1)	2.67 (0.25–28.3)	0.416
Race					
White/Caucasian	24 (68.6)	11 (57.9)	13 (81.3)	1.00	
All others	11 (31.4)	8 (42.1)	3 (18.8)	0.32 (0.07–1.50)	0.147
Marital status					
Single	23 (62.2)	10 (50.0)	13 (76.5)	1.00	
Married	14 (37.8)	10 (50.0)	4 (23.5)	0.31 (0.07–1.28)	0.104
Education					
4-year degree	2 (8.3)	1 (6.7)	1 (11.1)	1.00	
HS diploma or equivalent	22 (91.7)	14 (93.3)	8 (88.9)	0.57 (0.03–10.44)	0.706
Military rank					
Junior enlisted (E1-E3)	22 (66.7)	9 (52.9)	13 (81.3)	1.00	
NCO (E4-E5)	11 (33.3)	8 (47.1)	3 (18.8)	0.26 (0.5–1.26)	0.094
**Referral source**					
Self-referred	19 (50.0)	13 (61.9)	6 (35.3)	1.00	
Superiors encouraged	14 (36.8)	6 (28.6)	8 (47.1)	2.89 (0.69–12.12)	0.147
Command-directed	5 (13.2)	2 (9.5)	3 (17.7)	3.25 (0.43–24.84)	0.256
**Clinical characteristics ^1^**					
Inpatient history					
No	32 (91.4)	18 (94.7)	14 (87.5)	1.00	
Yes	3 (8.6)	1 (5.3)	2 (12.5)	2.57 (0.21–31.23)	0.459
Outpatient history					
No	21 (60.0)	10 (52.6)	11 (68.8)	1.00	
Yes	14 (40.0)	9 (47.4)	5 (31.3)	0.51 (0.13–2.03)	0.335
History of SI/SA					
No	26 (68.4)	16 (76.2)	10 (58.8)	1.00	
Yes	12 (31.6)	7 (41.2)	5 (23.8)	2.24 (0.56–9.02)	0.257
Diagnostic category					
No diagnosis	2 (5.3)	1 (4.8)	1 (5.9)	1.00	
V-code	2 (5.3)	2 (9.5)	0	-	
Adjustment disorder	7 (18.4)	4 (19.1)	3 (17.7)	0.75 (0.03–17.51)	0.858
Other axis-I disorder	27 (71.1)	14 (66.7)	13 (76.5)	0.93 (0.05–16.42)	0.960

* Data presented as No. (%), except as noted. ^1^ Due to unreported data, some variables will not have the full sample of 38 participants.

**Table 3:** Phase 2: Results of conditional logistic regression predicting career-related outcomes among treatment-seeking and non-treatment-seeking U.S. Marine Corps service members (*N* = 178) *.

Career-Related Variables	Total	Treatment-Seeking	Control	Conditional Model ^2^	
	(*N* = 178)	(*n* = 40)	(*n* = 138)	OR (95% CI)	*p*
Record status					
Active record	47 (26.4)	2 (5.0)	45 (32.6)	1.00	-
Separated	125 (70.2)	38 (95.0)	87 (63.0)	10.16 (2.30–44.91)	0.002
On orders	5 (2.8)	0	5 (3.6)	-	0.986
Separation code ^1^					
Voluntary	26 (20.8)	13 (34.2)	13 (14.9)	1.00	-
Involuntary	44 (35.2)	15 (39.5)	29 (33.3)	0.42 (0.13–1.41)	0.159
Completion of service	55 (44.0)	10 (26.3)	45 (51.7)	0.19 (0.05–0.66)	0.010
Security classification					
Secret/top secret/SCI	131 (73.6)	26 (65.0)	105 (76.1)	1.00	-
Denied	2 (1.1)	1 (2.5)	1 (0.7)	5.11 (0.30–87.42)	0.261
No status	39 (21.9)	10 (25.0)	29 (21.0)	1.50 (0.61–3.70)	0.374
Revoked/loss of jurisdiction	6 (3.4)	3 (7.5)	3 (2.2)	3.79 (0.71–20.29)	0.120
Legal action					
No	127 (71.4)	22 (55.0)	105 (76.1)	1.00	-
Yes	51 (58.7)	18 (45.0)	33 (23.9)	2.84 (1.32–6.24)	0.008
Average time to separation, mean (SD), years	3.3 (2.2)	1.5 (1.5)	3.8 (2.1)	-	<0.001

* Data presented as No. (%), except as noted. ^1^ Due to unreported data, some variables will not have the full sample of 178 participants. ^2^ Logistic regression results where a cluster variable was created to account for the matched case: control study design. Note. SCI = sensitive compartmented information.
